# Transverse Incision for Pancreatoduodenectomy Reduces Wound Complications: A Single-Center Analysis of 399 Patients

**DOI:** 10.3390/jcm12082800

**Published:** 2023-04-10

**Authors:** Stefanie Junker, Anne Jacobsen, Susanne Merkel, Axel Denz, Christian Krautz, Georg F. Weber, Robert Grützmann, Maximilian Brunner

**Affiliations:** Department of General and Visceral Surgery, Friedrich-Alexander-University, Krankenhausstraße 12, 91054 Erlangen, Germany; stefanie.junker@uk-erlangen.de (S.J.); anne.jacobsen@uk-erlangen.de (A.J.); susanne.merkel@uk-erlangen.de (S.M.); axel.denz@uk-erlangen.de (A.D.); christian.krautz@uk-erlangen.de (C.K.); georg.weber@uk-erlangen.de (G.F.W.); robert.gruetzmann@uk-erlangen.de (R.G.)

**Keywords:** pancreatoduodenectomy, midline incision, transverse incision, fascial dehiscence, surgical site infection, incisional hernia

## Abstract

Background: Even if the minimally invasive approach is advancing in pancreatic surgery, the open approach is still the standard for a pancreatoduodenectomy. There are two types of incisions used: the midline incision (MI) and transverse incision (TI). The aim of this study was to compare these two incision types, especially regarding wound complications. Methods: A retrospective review of 399 patients who underwent a pancreatoduodenectomy at the University Hospital Erlangen between 2012 and 2021 was performed. A total of 169 patients with MIs were compared with 230 patients with TIs, with a focus on postoperative fascial dehiscence, postoperative superficial surgical site infection (SSSI) and the occurrence of incisional hernias during follow-up. Results: Postoperative fascial dehiscence, postoperative SSSI and incisional hernias occurred in 3%, 8% and 5% of patients, respectively. Postoperative SSSI and incisional hernias were significantly less frequent in the TI group (SSI: 5% vs. 12%, *p* = 0.024; incisional hernia: 2% vs. 8%, *p* = 0.041). A multivariate analysis confirmed the TI type as an independent protective factor for the occurrence of SSSI and incisional hernias (HR 0.45 (95% CI = 0.20–0.99), *p* = 0.046 and HR 0.18 (95% CI = 0.04–0.92), *p* = 0.039, respectively). Conclusion: Our data suggest that the transverse incision for pancreatoduodenectomy is associated with reduced wound complications. This finding should be confirmed by a randomized controlled trial.

## 1. Introduction

Minimally invasive approaches have gained increasing popularity in pancreatic surgery. However, the open approach might be the standard for a pancreatoduodenectomy at the current time. A pancreatoduodenectomy is associated with relevant morbidity and even mortality [1,2,3,4]. Wound complications represent a relevant proportion of morbidity, especially in open surgery, and contribute to a longer length of hospital stay, the need for readmissions and resurgery as well as significant costs. In the literature, the rates of superficial surgical site infection (SSSI) after a pancreatoduodenectomy mostly vary between 8.2% and 13.4% [4,5,6,7]. The occurrence of incisional hernias after a pancreatoduodenectomy has been reported in 17.7% of patients [8].

Therefore, there is an ongoing debate about several aspects potentially influencing the occurrence of postoperative wound complications. In the literature, the focus is particularly on the type of antibiotic prophylaxis, suture technique and suture material [9,10,11]. Moreover, the type of incision is an unsolved topic of discussion [12,13,14,15,16,17,18,19,20]. The most common types of incisions for an open pancreatoduodenectomy are the midline incision (MI) and the transverse incision (TI). Proponents of the midline incision argue that a midline laparotomy represents the more universal approach, especially if multivisceral resection is necessary, and an extension of the incision can be carried out more easily. In addition, no abdominal wall muscles need to be severed, so access trauma is considered to be less. Moreover, wound opening and closure are more time-sparing during midline incisions. On the opposite side, the transverse incision (TI) is said to have an excellent lateral entry point for the surgeon and may be associated with a lower rate of incisional hernias. However, the current standard might be that the choice of incision is primarily based on surgeon preference.

Previous studies comparing these two open approaches are limited and show heterogeneous results [12,13,14,15,16,17,18,19,20]. Moreover, all these studies included heterogeneous patient collectives (partially emergency and elective surgery, or surgery for different indications). Until now, there has been no study comparing these two approaches in a homogeneous cohort during pancreatic surgery.

The aim of the present study was to compare midline incisions (MIs) and transverse incisions (TIs) during pancreatoduodenectomies, regarding wound complications, especially the occurrence of postoperative superficial surgical site infection (SSSI), postoperative fascial dehiscence and incisional hernias during follow-up.

## 2. Materials and Methods

We retrospectively analyzed 399 consecutive patients who underwent open pancreatoduodenectomies from 2012 to 2021 at the University Hospital Erlangen. Any indications for the pancreatoduodenectomy were allowed. Patients who underwent minimally invasive pancreatoduodenectomies were excluded.

Data on the patient demographics, comorbidities, pre- and intraoperative parameters and surgical incision technique as well as on the postoperative course, including short- and long-term wound complications, were obtained and analyzed. The primary outcomes were the occurrence of fascial dehiscence, superficial surgical site infection (SSSI) and incisional hernias (see definitions below). Patients were stratified into two groups: (1) patients with midline incisions (MI); and (2) patients with transverse incisions (TIs). The recorded parameters were compared between these two groups.

This study was approved by the Ethics Committee of FAU Erlangen (22-165-Br).

### 2.1. Definitions

Postoperative fascial dehiscence was defined as a relevant gap between the margins of the fascia during the hospital stay resulting in resurgery. Postoperative superficial surgical site infection was defined according to the wound healing CDC definition for superficial incisional SSI [21]. An incisional hernia was defined as a clinically and/or sonographically clearly identified hernia during follow-up reconsultations.

Postoperative pancreatic fistula (POPF), delayed gastric emptying (DGE) and postpancreatectomy hemorrhage (PPH) were defined according to the definition of the International Study Group of Pancreatic Fistula (ISGPF). POPF grades B and C were considered to be clinically relevant POPF (CR-POPF) [22,23,24,25].

### 2.2. Surgical Techniques

All patients received preoperative intravenous antibiotic prophylaxis with cephalosporin and metronidazole. The skin was washed with an antiseptic solution. The skin incision was performed with a conventional scalpel. The midline laparotomy was performed on the median line, circumcising the umbilicus. The transverse laparotomy was performed in a curved way, slightly to the right. The subcutaneous tissue and the fascia were divided using a monopolar scalpel. The incised abdominal wall was covered with abdominal cloths during the operation. Fascia closure was always performed using a monofilament absorbable PDS 1 loop suture. Midline incisions were closed with a single-layer technique whereas transverse incisions were closed with a double-layer technique. A subcutaneous drain was always inserted. The skin closure was performed using standard skin staples.

The decision about the incision technique was made by the surgeon, depending on his or her preference or on the anatomical conditions of the costal arch.

### 2.3. Statistical Analysis

The data analysis was performed with SPSS software (SPSS, version 28.0). Comparisons of the metric and ordinal data were calculated with a Student’s *t*-test or the Mann–Whitney U test. The chi-squared test was used for the categorical data. The statistical significance was set at *p* < 0.05. For the analysis of the occurrence of incisional hernias, only patients without in-hospital mortality and with a minimal follow-up of 12 months were included (*n* = 249). The reasons for exclusion were in-hospital mortality in 15 (10%) patients, 12 month mortality (excluding in-hospital mortality) in 79 (53%) and lost to follow-up in 56 (37%). All recorded parameters were tested as potential risk factors for wound complications (fascial dehiscence, SSSI and incisional hernias) using a univariate analysis. For the metric parameters, the median was chosen as the cutoff. Associations with wound complications with a *p*-value < 0.05 in the univariate analysis were included in the multivariate analysis using a logistic regression. The probability curve of the occurrence of an incisional hernia was plotted using the Kaplan–Meier method and compared with the log-rank test.

## 3. Results

### 3.1. Demographics

Of the 399 patients (median age: 69 years, 40% female) who underwent a pancreatoduodenectomy during the study period, a midline incision was applied in 169 patients (MI group) and a transverse incision in 230 patients (TI group). All demographic parameters, including age, sex, ASA, BMI, comorbidities and preoperative blood results, did not significantly differ between the groups (Table 1).

### 3.2. Surgical Parameters

Pancreatic carcinomas (48%), chronic pancreatitis (12%), cholangiocarcinomas (10%) and ampullary carcinomas (9%) were the most common indications for surgery. A pancreatic head resection was performed in 58% as a pylorus-preserving pancreatoduodenectomy (PPPD) and in 42% as a Whipple procedure. An additional multivisceral resection was necessary in 12% of the patients. A transverse incision was used significantly more often for PPPD; vice versa, a midline incision was used more often for the Whipple procedure (*p* < 0.001). All other surgical parameters showed no significant difference between the MI and TI groups (Table 2).

### 3.3. Outcome Parameters

Regarding general in-hospital morbidity, the overall morbidity and mortality in our cohort were 59% and 4%, respectively. CR-POPF, DGE and PPH occurred in 18%, 27% and 7%, respectively. A total of 13% of the patients required resurgery. The median length of postoperative stay was 18 days. The use of a transverse incision (TI) was associated with significantly less morbidity, less CR-POPF, a lower resurgery rate and a shorter length of postoperative stay compared with the MI group (51% vs. 69%, *p* < 0.001; 12% vs. 25%, *p* = 0.001; 9% vs. 18%, *p* = 0.006; 21 days vs. 15 days, *p* < 0.001) (Table 3).

**Figure 1:**
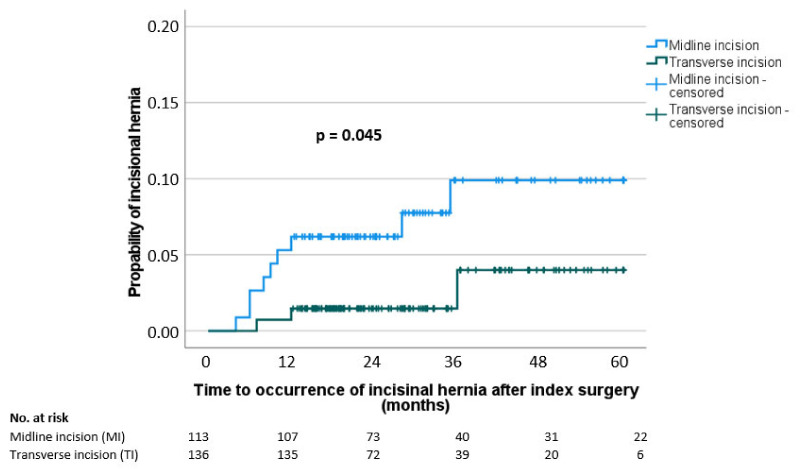
Probability of incisional hernia stratified by incision type (midline incision (MI) and transverse incision (TI)) (*n* = 249).

Regarding postoperative wound complications, the rates of fascia dehiscence and superficial surgical site infection (SSSI) were 3% and 8%, respectively. Two patients (1%) needed a surgical revision due to SSSI. During the follow-up (median 32.4 months), an incision hernia was diagnosed in 5% of the patients. SSSI and incisional hernias occurred significantly less frequently in the TI group (5% vs. 12%, *p* = 0.024; 2% vs. 8%, *p* = 0.041) whereas there was no significant difference in the occurrence of fascial dehiscence between the groups (2% vs. 3%, *p* = 1.000) (Table 3). The probability curve of the occurrence of an incisional hernia showed a risk of an incisional hernia at the 5 year follow-up of 4% in the transverse incision group and of 10% in the midline incision group (*p* = 0.045) (Figure 1).

### 3.4. Risk Factors for Wound Complications

Regarding the occurrence of postoperative fascial dehiscence, we identified three significant risk factors in the univariate analysis: the presence of COPD (*p* = 0.045); a preoperative CRP level above 5 mg/L (*p* = 0.024); and the occurrence of PPH (*p* = 0.003). Among these variables, only the occurrence of PPH (HR 7.84 (95% CI = 1.94–31.76), *p* = 0.004) was confirmed as an independent risk factor for the development of fascial dehiscence in the multivariate analysis (Table 4).

In the univariate analysis, five risk factors were significantly associated with a postoperative SSSI: a preoperative CRP level above 5 mg/L (*p* = 0.002); the use of a midline incision (*p* = 0.045); an operative time of more than 300 min (*p* = 0.047); an intraoperative blood loss of more than 500 mL (*p* = 0.040); and the need for postoperative resurgery before the occurrence of a wound complication (*p* = 0.003). The multivariate analysis revealed the use of a midline incision (HR 2.24 (95% CI = 1.02–4.95), *p* = 0.046) and a preoperative CRP level above 5 mg/L (HR 3.43 (95% CI = 1.34–8.77), *p* = 0.002) as independent risk factors for postoperative SSI (Table 4).

For the occurrence of an incisional hernia, five risk factors were identified: the presence of COPD (*p* = 0.045); a preoperative CRP level above 5 mg/L (*p* = 0.005); the use of a midline incision (*p* = 0.041); and the postoperative occurrence of fascial dehiscence (*p* = 0.029) or SSI (*p* = 0.046). The multivariate analysis, including the mentioned factors, confirmed the use of a midline incision (HR 5.49 (95% CI = 1.09–27.78), *p* = 0.039), the presence of COPD (HR 24.39 (95% CI = 1.64–391.94), *p* = 0.020), a preoperative CRP level above 5 mg/L (HR 11.43 (95% CI = 1.27–103.15), *p* = 0.030) and the occurrence of postoperative fascial dehiscence (HR 14.11 (95% CI = 1.10–180.44), *p* = 0.042) as independent risk factors for the occurrence of incisional hernias (Table 4).

## 4. Discussion

Wound complications play a relevant role that should not be underestimated in the postoperative course of pancreatoduodenectomies, which are prone to complications. Our data showed that in the context of open pancreatoduodenectomies, a transverse incision had significant advantages compared with a midline laparotomy regarding the occurrence of superficial surgical site infection and incisional hernias.

The rates of fascial dehiscence (3%) and SSSI (8%) in our cohort were in line with previously reported rates after a pancreatoduodenectomy [4,5,6,7]. The occurrence of incisional hernias in our cohort (5%) was slightly lower than that reported in the literature [8,12,13]. This might be explained by a partially shorter median follow-up in our cohort than in other studies. However, our median follow-up was 32 months, which is the period when the majority of incisional hernias occur.

Previous studies on the best incision type in abdominal surgery are heterogeneous and show ambiguous results. A systematic review from 2013 identified 17 randomized trials comparing transverse and midline incisions. In this review, there was no difference in wound dehiscence and wound infections between the two groups, but there was a significantly lower incisional hernia rate in the transverse incision group (seven trials included > TI: 4.5% vs. MI: 9.9%; RR 1.77 (95% CI = 1.09–2.87, *p* = 0.02) [14]. However, the comparability of these studies is limited due to several aspects; the mentioned studies investigated heterogeneous study cohorts (cholecystectomies, aortoiliac surgery, right hemicolectomy, general abdominal surgeries and others), had partially small sample sizes (minimum 19 patients), included partially elective and emergency surgery, had partially short follow-ups (minimum 4.4 month median), used partially different definitions of the wound endpoints and examined a wide variety of outcome parameters (next to wound complications, especially pulmonary complications, OR time, pain level and others).

Regarding only current (from 2000 to today) randomized trials including at least partially pancreatic resections, only two studies could be identified [12,16]. In the trial of Proske et al. (94 patients, 22% pancreatic resections), there was no difference in postoperative wound complications between the groups (TI: 7% vs. MI: 8%, *p* = 1.000). The hernia rate was not investigated in this trial [16]. The POVATI trial (191 patients, 45% pancreatic resections) showed more wound infections in the TI group (TI: 15% vs. MI: 5%, *p* = 0.02) and no difference between the groups regarding the occurrence of a postoperative burst abdomen (TI: 1% vs. MI: 0%, *p* = 0.50) or an incisional hernia after one year (TI: 12% vs. MI: 16%, *p* = 0.48) [12]. Therefore, these results differed from those in our cohort. Possible explanations for these differences were, again, different inhomogeneous study cohorts, smaller sample sizes and different primary study endpoints. However, our results regarding the lower SSI rate in the TI group were in line with a current retrospective study (696 patients, 23% pancreatic resections), which compared transverse, midline and modified Makuuchi incisions for different abdominal surgeries (TI: 17% vs. MI: 28%, *p* = 0.04) [13].

Our study was the first analysis using a homogeneous cohort of patients undergoing pancreatoduodenectomies, which was a relevant strength of our analysis. However, the present study had several limitations. First, the retrospective design of our study may have incurred bias. Second, our TI group was associated with significantly less morbidity, less CR-POPF and a lower resurgery rate. Subsequently, the differences in wound complications between the incision groups could be affected by these factors; on the one hand, through a potential reduction in wound complications as a subsequent complication of other occurred complications and, on the other hand, through potentially generally fewer complications during the operation of surgeons preferring a transverse incision. However, morbidity, CR-POPF and resurgery were included in the multivariate analysis and showed no significant influence. In our study, there remained the potential for surgeon bias as the operating surgeon chose their preferred technique without the randomization of the incision technique. This resulted in the majority of patients undergoing a pancreatoduodenectomy at the beginning of the study period with a midline incision and, towards the end of the study period, most patients received a transverse incision. This sequence of events again represented a potential source of bias in the study. Third, we included all patients with a follow-up of at least 12 months to obtain statistical power through a relevant number of patients. In addition, the drop-out rate during follow-up was quite high. This could be explained by the inclusion of patients with a maximum follow-up time of 12 months and the investigated patient cohort per se, which included a high proportion of patients with pancreatic carcinomas. These aspects and the median follow-up of 32 months in our cohort could lead to a lower rate of incisional hernias, thus potentially distorting the results.

## 5. Conclusions

Our results suggested that a transverse incision for a pancreatoduodenectomy was associated with a significantly lower SSSI and incisional hernia rate. Randomized controlled trials during pancreatic resections are needed to confirm these findings.

## Figures and Tables

**Table 1:** Characteristics of patients undergoing pancreatoduodenectomy (*n* = 399) stratified by incision type (MI (midline incision) vs. TI (transverse incision)).

	MI	TI	*p*-Value
**Number**	169	230	
**Age (years), median (IQR)**	68 (19)	69 (18)	0.768
**Gender, *n* (%)**			0.353
**Female**	72 (43)	87 (38)	
**Male**	97 (57)	143 (62)	
**ASA, *n* (%)**			0.231
**I**	6 (4)	5 (2)	
**II**	98 (58)	114 (50)	
**III**	64 (38)	110 (48)	
**IV**	1 (1)	1 (0)	
**BMI (kg/m^2^), median (IQR)**	25.9 (6.0)	25.4 (5.4)	0.315
**Active alcohol abuse, *n* (%)**	14 (8)	11 (5)	0.209
**Active nicotine abuse, *n* (%)**	41 (24)	52 (23)	0.720
**Nutritional risk score (NRS), *n* (%)**			0.218
**≤3**	138 (82)	175 (76)	
**>3**	31 (18)	55 (24)	
**Comorbidity, *n* (%)**			
**Hypertension**	84 (50)	135 (59)	0.084
**Diabetes**	45 (27)	68 (30)	0.574
**Cardiovascular**	24 (14)	39 (17)	0.490
**COPD**	4 (2)	10 (4)	0.411
**Cerebrovascular**	7 (4)	5 (2)	0.375
**Previous abdominal surgery, *n* (%)**	73 (43)	103 (45)	0.761
**Preoperative chemotherapy or chemoradiation, *n* (%)**	24 (14)	22 (10)	0.157
**Preoperative WBC (10^9^/L), median (IQR)**	6.9 (2.8)	7.3 (2.6)	0.547
**Preoperative albumin (g/L), median (IQR)**	39.4 (8.1)	39.6 (7.2)	0.485
**Preoperative CRP (mg/L), median (IQR)**	6 (13)	5 (11)	0.693

ASA: American Society of Anesthesiologists’ classification; BMI: body mass index; COPD: chronic obstructive pulmonary disease; WBC: white blood cells; CRP: C-reactive protein.

**Table 2:** Surgical details of patients undergoing pancreatoduodenectomy (*n* = 399) stratified by incision type (MI (midline incision) vs. TI (transverse incision)).

	MI (*n* = 169)	TI (*n* = 230)	*p*-Value
**Indication for surgery, *n* (%)**			-
**Pancreatic carcinoma**	68 (40)	124 (54)	
**Ampullary carcinoma**	27 (16)	8 (4)	
**Cholangiocarcinoma**	20 (12)	21 (9)	
**Duodenal carcinoma**	2 (1)	7 (3)	
**Neuroendocrine neoplasm**	11 (7)	11 (5)	
**Chronic pancreatitis**	11 (7)	35 (15)	
**Cystic pancreatic lesion**	7 (4)	15 (7)	
**Other indication**	23 (14)	9 (4)	
**Type of surgery, *n* (%)**			**<0.001**
**Whipple procedure**	116 (69)	52 (23)	
**PPPD**	53 (31)	178 (77)	
Multivisceral resection *, *n* (%)	25 (15)	24 (10)	0.218
**Operative time (min), median (IQR)**	295 (94)	318 (99)	0.082
**Intraoperative blood loss (mL), median (IQR)**	500 (550)	500 (500)	0.099
**Intraoperative fluids, median (IQR)**	5500 (3025)	5200 (2775)	0.437

PPPD: pylorus-preserving pancreaticoduodenectomy. * including additional small bowel, colon, gastric and liver resections. Bold *p*-value = significant.

**Table 3:** Outcome parameters of patients undergoing pancreatoduodenectomy (*n* = 399 and 249, respectively) stratified by incision type (MI (midline incision) vs. TI (transverse incision)).

	MI (*n* = 169)	TI (*n* = 230)	*p*-Value
***In-hospital general morbidity***			
**Morbidity, *n* (%)**	116 (69)	118 (51)	**<0.001**
**Mortality, *n* (%)**	6 (4)	9 (4)	1.000
**Reoperation, *n* (%)**	31 (18)	20 (9)	**0.006**
**CR-POPF, *n* (%)**	42 (25)	28 (12)	**0.001**
**DGE, *n* (%)**	53 (31)	55 (24)	0.111
**PPH, *n* (%)**	12 (7)	16 (7)	1.000
**Length of postoperative stay (days), median (IQR)**	21 (18)	15 (12)	**<0.001**
***In-hospital wound complications***			
**Fascial dehiscence, *n* (%)**	4 (2)	6 (3)	1.000
**Superficial surgical site infection (SSSI), *n* (%)**	20 (12)	12 (5)	**0.024**
**Reoperation due to SSSI, *n* (%)**	1 (1)	1 (0)	1.000
Long-term wound complications *			
**Follow-up for hernia occurrence (months), median (IQR)**	35.5 (34.4)	32.8 (22.6)	0.061
**Incision hernia, *n* (%)**	9 (8)	3 (2)	**0.041**

CR-POPF: clinically relevant postoperative pancreatic fistula; DGE: delayed gastric emptying; PPH: postpancreatectomy hemorrhage; SSI: surgical site infection. * patients without in-hospital mortality and with a minimal follow-up of 12 months > *n* = 249. Bold *p*-value = significant.

**Table 4:** Risk factors for postoperative fascial dehiscence, SSI and incisional hernias in patients undergoing pancreatoduodenectomy (*n* = 399 and 249, respectively).

	Fascial Dehiscence				SSSI				Incisional Hernia *			
	Univariate	Multivariate			Univariate	Multivariate			Univariate	Multivariate		
		HR	95% CI	*p*-Value		HR	95% CI	*p*-Value		HR	95% CI	*p*-Value
**Age (** **≤70 vs. >70 years)**	0.109	-	-	-	0.139	-	-	-	0.224	-	-	-
**Gender (female vs. male)**	0.327	-	-	-	0.350	-	-	-	0.371	-	-	-
**ASA (I/II vs. III/IV)**	0.347	-	-	-	0.853	-	-	-	0.766	-	-	-
**BMI (≤25 vs. >25 kg/m^2^)**	0.762	-	-	-	0.586	-	-	-	0.150	-	-	-
**Active alcohol abuse (yes vs. no)**	1.000	-	-	-	0.514	-	-	-	0.124	-	-	-
**Active nicotine abuse (yes vs. no)**	0.704	-	-	-	0.664	-	-	-	0.712	-	-	-
**NRS (** **≤3 vs. >3)**	1.000	-	-	-	0.504	-	-	-	0.274	-	-	-
**Hypertension (yes vs. no)**	0.763	-	-	-	0.266	-	-	-	0.772	-	-	-
**Diabetes (yes vs. no)**	1.000	-	-	-	1.000	-	-	-	0.311	-	-	-
**Cardiovascular (yes vs. no)**	0.374	-	-	-	1.000	-	-	-	0.071	-	-	-
**COPD (yes vs. no)**	**0.045**	5.1	0.8–31.4	0.078	0.614	-	-	-	**0.045**	**24.4**	**1.6–391.9**	**0.020**
**Cerebrovascular (yes vs. no)**	1.000	-	-	-	1.000	-	-	-	1.000	-	-	-
**Previous abdominal surgery (yes vs. no)**	0.114	-	-	-	0.578	-	-	-	1.000	-	-	-
**Neoadjuvant therapy (yes vs. no)**	0.386	-	-	-	0.406	-	-	-	0.643	-	-	-
**Preop. WBC (≤7 vs. >7 × 10^9^/L)**	0.343	-	-	-	0.141	-	-	-	1.000			
**Preop. Albumin (≤40 vs. >40 g/L)**	0.195	-	-	-	0.077	-	-	-	0.367			
**Preop. CRP (≤5 vs. >5 mg/L)**	**0.024**	5.8	0.7–47.4	0.104	**0.002**	**3.4**	**1.3–8.8**	**0.010**	**0.005**	**11.4**	**1.3–103.2**	**0.030**
**Type of surgery (Whipple vs. PPPD)**	0.748	-	-	-	0.197	-	-	-	0.135	-	-	-
**Multivisceral resection (yes vs. no)**	0.510	-	-	-	0.341	-	-	-	0.545	-	-	-
**Type of incision (MI vs. TI)**	1.000	-	-	-	**0.024**	**2.2**	**1.0–5.0**	**0.046**	**0.041**	**5.5**	**1.1–27.8**	**0.039**
**Operative time (≤300 vs. >300 min)**	0.758	-	-	-	**0.047**	2.0	0.9–4.8	0.101	1.000	-	-	-
**Intraop. blood loss (** **≤500 vs. >500 mL)**	0.754	-	-	-	**0.040**	1.5	0.7–3.2	0.371	0.370	-	-	-
Reoperation (yes vs. no) **	0.609	-	-	-	**0.003**	2.4	1.0–6.0	0.053	0.613	-	-	-
**CR-POPF (yes vs. no)**	0.390	-	-	-	0.330	-	-	-	0.243	-	-	-
**DGE (yes vs. no)**	0.143	-	-	-	0.212	-	-	-	1.000	-	-	-
**PPH (yes vs. no)**	**0.003**	**7.8**	**1.9–31.8**	**0.004**	0.265	-	-	-	1.000	-	-	-
**Fascial dehiscence (yes vs. no)**	-	-	-	-	-	-	-	-	**0.029**	**14.1**	**1.1–180.4**	**0.042**
**Superficial surgical site infection (yes vs. no)**	-	-	-	-	-	-	-	-	**0.046**	0.9	0.1–9.6	0.921

SSSI: surgical site infection; ASA: American Society of Anesthesiologists’ classification; BMI: body mass index; NRS: nutritional risk score; COPD: chronic obstructive pulmonary disease; preop.: preoperative; WBC: white blood cells; CRP: C-reactive protein; MI: midline incision; TI: transverse incision; intraop.: intraoperative; CR-POPF: clinically relevant postoperative pancreatic fistula; DGE: delayed gastric emptying; PPH: postpancreatectomy hemorrhage. * Patients without in-hospital mortality and with a minimal follow-up of 12 months > *n* = 249; ** before the occurrence of wound complications. Bold *p*-value = significant.

## Data Availability

All data generated or analyzed during this study are included in this published article.
